# Green Synthesis of Silver Nanoparticles From *Macaranga tanarius* (L.) Mull. Arg. Leaf Extract With Enhanced Antibacterial, Antioxidant, and Tyrosinase Inhibitory Activities

**DOI:** 10.1155/ijm/3943779

**Published:** 2026-07-30

**Authors:** Hsin-Ya Yeh, Mohit Chand, Soma Sardar Barawi, Han-Chen Ho, Chia-Jyi Liu, Chih-Wei Chang, Higuchi Kazuki, Jui-Yu Chou

**Affiliations:** ^1^ Department of Biology, National Changhua University of Education, Changhua, Taiwan, ncue.edu.tw; ^2^ International Program for Master of Science in Materials and Biological Technology, and Science Education, National Changhua University of Education, Changhua, Taiwan, ncue.edu.tw; ^3^ Faculty of Computer Science, Dalhousie University, Halifax, Canada, dal.ca; ^4^ Electron Microscopy Laboratory, Department of Anatomy, Tzu Chi University, Hualien, Taiwan, tcu.edu.tw; ^5^ Department of Physics, National Changhua University of Education, Changhua, Taiwan, ncue.edu.tw; ^6^ Department of Chemistry, National Changhua University of Education, Changhua, Taiwan, ncue.edu.tw; ^7^ Atopic Dermatitis Treatment Center, Osaka, Japan

**Keywords:** antibacterial activity, atopic dermatitis (AD), *Macaranga tanarius*, silver nanoparticles, *Staphylococcus aureus*

## Abstract

Atopic dermatitis (AD) is a chronic inflammatory skin disorder characterized by microbial imbalance, particularly the overgrowth of *Staphylococcus aureus*. This study reports the green synthesis of silver nanoparticles (AgNPs) using *Macaranga tanarius* extract (MTE) and evaluates their in vitro antibacterial and bioactive properties. The synthesized MT‐AgNPs exhibited a distinct surface plasmon resonance (SPR) peak at 430 nm, with TEM revealing predominantly spherical morphologies (mean size: 29.2 nm; range: 6–87 nm). Phytochemical analysis confirmed that MTE is rich in polyphenols (2429 ± 33.7 mg GAE/100 g) and flavonoids (236.3 ± 10.7 mg QE/100 mL), contributing to its strong antioxidant capacity (82.72*%* ± 3.12*%* DPPH scavenging; FRAP: 20.36 ± 0.02 mg FeSO_4_/100 mL). MT‐AgNPs demonstrated potent antibacterial activity against 17 clinical *S. aureus* isolates (e.g., *mecA*‐positive strains), with MICs of 9.375–18.75 *μ*g/mL. At 6.25 *μ*g/mL, MT‐AgNPs significantly inhibited biofilm formation by up to 63% (*p* < 0.05). Crucially, MTE displayed a highly stable tyrosinase inhibitory profile, maintaining a consistent inhibition rate of approximately 45% throughout a 120‐min kinetic assay (equivalent to 6.0–8.5 mg kojic acid/g). This sustained inhibitory stability suggests its potential application in addressing postinflammatory hyperpigmentation. These findings indicate that MT‐AgNPs serve as a promising, eco‐friendly antibacterial nanomaterial with potential in vitro applications for managing AD‐related microbial challenges and skin recovery.

## 1. Introduction

The skin microbiome is a complex and dynamic ecosystem whose dysbiosis is central to the pathogenesis of various inflammatory skin disorders, including atopic dermatitis (AD). AD is a multifactorial disease characterized by genetic defects, skin barrier dysfunction, immune imbalances, and microbial dysbiosis [1]. Patients often experience chronic inflammation and an increased risk of infection [2]. Specifically, reduced filaggrin levels and decreased antimicrobial peptide production foster an environment conducive to the overgrowth of pathogenic bacteria such as *Staphylococcus aureus*, resulting in decreased microbial diversity [3]. Metabolites from these pathogens can penetrate the skin barrier, exacerbating inflammation and altering immune responses [4]. Current treatments primarily involve corticosteroids, which provide rapid symptom relief but do not address the underlying condition and pose risks of side effects with prolonged use [5]. Therefore, exploring alternative, biocompatible therapies is crucial.

Plants contain a diverse array of bioactive compounds that provide promising complementary approaches to conventional treatments [6, 7]. *Macaranga tanarius* (L.) Mull. Arg. is a woody plant traditionally employed in ethnomedicine for treating various skin‐related ailments, including cuts, burns, and ulcers [8, 9]. These medicinal applications are attributed to its rich phytochemical profile, including prenylated flavonoids, tannins, stilbenoids, and terpenoids [10–12]. These compounds are associated with antioxidant, anti‐inflammatory, and antimicrobial properties [13]. However, despite the known bioactivity of *M. tanarius*, its potential in synthesizing AgNPs to target the pathogenic microbial imbalance specific to AD patients—including the inhibition of clinical *S. aureus*—remains to be rigorously evaluated.

Silver‐containing antimicrobial agents have a long history of use in medical dressings and food packaging [14, 15]. Silver nanoparticles (AgNPs) have garnered significant attention due to their high biocompatibility and efficacy against multidrug‐resistant (MDR) strains [16, 17]. In the context of AD, silver was selected for its potent, multitargeted antibacterial mechanism, which disrupts bacterial cell walls, proteins, and DNA simultaneously, minimizing resistance risks [18, 19]. While traditional synthesis methods often involve toxic agents, green synthesis routes significantly reduce ecological footprints [20, 21]. Furthermore, plant‐based bioactive compounds provide a unique capping layer that enhances the stability and biocompatibility of nanoparticles, which is critical for medical applications [22]. Leveraging the polyphenolic profile of *M. tanarius* offers a sustainable platform to target AD pathogens while maintaining low cytotoxicity [23].

Biosynthesis of AgNPs has emerged as a cost‐effective and eco‐friendly alternative to traditional physical and chemical approaches [24, 25]. Plant‐mediated synthesis leverages the abundance of natural antioxidant compounds, such as polyphenols, flavonoids, and terpenoids, which function as reducing agents to facilitate the reduction of silver ions (Ag^+^) from silver nitrate (AgNO_3_) into metallic AgNPs (Ag^0^) [26]. Additionally, plant‐derived biomolecules, including proteins and organic acids, serve as effective capping and stabilizing agents that enhance the biological activity of the resulting nanoparticles [24, 25].

While the phytochemical properties of *M. tanarius* have been documented [8], its application as a precursor for AgNP synthesis remains an unexplored avenue in the development of targeted therapies for AD. Given the increasing prevalence of antibiotic‐resistant *S. aureus* in AD patients, there is an urgent need to evaluate biogenic nanomaterials that offer both potent antibacterial action and high biocompatibility. Consequently, this study was designed to systematically evaluate the synergistic pharmacological potential of *M. tanarius*–mediated AgNPs (MT‐AgNPs). We hypothesized that the integration of *M. tanarius* bioactive compounds—specifically its antioxidant and tyrosinase inhibitory metabolites—onto the silver core would yield an agent with potent antibacterial efficacy tailored to the microbial dysbiosis of AD skin. To test this, our specific objectives were to (1) characterize the phytochemical profile and bioactivities of *M. tanarius* extract (MTE), (2) synthesize and characterize MT‐AgNPs using a green route, and (3) evaluate the antibacterial and biofilm inhibitory efficacy of both MTE and MT‐AgNPs against clinical AD‐derived *S. aureus* strains. Furthermore, the antioxidant, tyrosinase inhibitory, and photocatalytic capabilities of MT‐AgNPs were assessed to evaluate their complementary roles in managing AD‐related skin conditions. This stepwise approach provides a coherent framework for developing targeted therapeutic strategies focused on addressing clinical *S. aureus* infections.

## 2. Materials and Methods

### 2.1. Sample Collection and Bacterial Isolation

Clinical samples were collected from 16 volunteers who were clinically diagnosed with AD under the supervision of a eczema specialist at ADLAB0358 Co., Ltd. (Osaka, Japan). The inclusion criteria for participant selection were (1) a confirmed clinical diagnosis of AD, (2) the presence of active inflammatory skin lesions, and (3) visible desquamation or scaling on the skin surface. Written informed consent was obtained from all participants after a clear explanation of the study objectives. To collect samples, sterile, dry cotton swabs were gently rubbed twice across the lesional skin surface of each participant. The swabs were stored at 4°C and subsequently streaked onto mannitol salt agar (MSA) plates (Bio Pioneer Tech Co., Ltd., Taiwan) for incubation at 37°C. A total of 81 bacterial isolates were recovered from these participants. To evaluate genetic diversity, RAPD analysis was performed in triplicate on all 81 isolates using the M13 primer (5 ^′^‐GAG GGT GGC GGT TCT‐3 ^′^) with sterile ddH_2_O as a negative control to ensure high reproducibility [27]. RAPD analysis was not conducted for isolates obtained from different individuals, which were presumed to be distinct strains. This comprehensive analysis ensured the evaluation of the distinct molecular profiles of all recovered strains.

For rapid genomic DNA extraction, 200 *μ*L of PEG‐NaOH solution was added to each sample in an eight‐strip PCR tube. The tubes were incubated at 80°C for 10 min in a PCR thermocycler (GeneAtlas G02, ASTEC, Fukuoka, Japan) to achieve cell lysis, followed by DNA amplification by PCR using the same system. Each 20 *μ*L PCR reaction mixture contained 2 *μ*L of M13 primer, 2 *μ*L of PuriTaq 2× PCR Master Mix (Cat. No. PU‐TQC‐100, Purigo), 2 *μ*L of denatured DNA, and sterile distilled water to a final volume of 20 *μ*L. The PCR cycling conditions included an initial denaturation at 95°C for 5 min, followed by 35 cycles of denaturation at 95°C for 30 s, annealing at 42°C for 30 s, and extension at 72°C for 90 s. A final extension step was carried out at 72°C for 10 min. PCR products were separated by electrophoresis on a 2% agarose gel using a SYBR Safe DNA Gel Stain (Thermo Fisher Scientific) at 110 V for 20–30 min. DNA bands were visualized under UV illumination, and isolates showing identical RAPD patterns were considered the same strain, with only one representative retained for further analysis.

For bacterial identification, universal primers 27F (5 ^′^‐AGA GTT TGA TCC TGG CTC AG‐3 ^′^) and 1541R (5 ^′^‐AAG GAG GTG ATC CAG CCG CA‐3 ^′^) were used to amplify the 16S rRNA gene [28, 29]. The PCR protocol consisted of an initial denaturation at 95°C for 5 min, followed by 35 cycles of denaturation at 95°C for 1 min, annealing at 55°C for 30 s, and extension at 72°C for 1 min and 45 s, with a final extension at 72°C for 10 min. PCR products were then subjected to electrophoresis on a 1% agarose gel containing a safe DNA stain, run at 110 V for 20–30 min. Positive samples were submitted for sequencing at Genomics, Inc. (New Taipei City, Taiwan). Species identification was conducted using the Basic Local Alignment Search Tool (BLAST) against the NCBI nucleotide database. To determine the presence of potential methicillin‐resistant *S. aureus* (MRSA), PCR detection of the *mecA* gene was performed using specific primers: mecA forward (5 ^′^‐GTA GAA ATG ACT GAA CGT CCG ATA A‐3 ^′^) and mecA reverse (5 ^′^‐CCA ATT CCA CAT TGT TTC GGT CTA A‐3 ^′^) [30]. The PCR amplification protocol included an initial denaturation at 94°C for 5 min, followed by 35 cycles of denaturation at 95°C for 45 s, annealing at 58°C for 45 s, and extension at 72°C for 45 s. A final extension was carried out at 72°C for 5 min. Amplified products (310 bp) were analyzed by electrophoresis on a 1.2% agarose gel with a safe DNA stain and visualized under UV light.

### 2.2. Preparation of MTE

*Macaranga tanarius* plant material was collected from the National Changhua University of Education, located in Changhua City, Taiwan (24.08272° N, 120.55838° E). Fresh leaves were thoroughly washed with tap water, followed by two rinses with deionized water to remove any surface contaminants. The leaves were then dried with paper towels and subsequently oven‐dried at 60°C for approximately 5 min to eliminate surface moisture. After drying, the leaves were cut into small pieces. A 10 g portion of the cut leaves was boiled in 100 mL of sterile deionized water for 15 min on a magnetic stirrer hot plate set at 250°C. Once cooled to room temperature, the extract was filtered through Whatman filter paper (Grade No. 1, 110 mm). For antibacterial testing, the MTE was further sterilized by filtration through a 0.22‐*μ*m syringe filter (Jet Bio‐Filtration Co., Ltd.). The sterile MTE was then stored at 4°C until needed for subsequent experiments.

### 2.3. Agar Well Diffusion Assay

The antibacterial activity of the samples was evaluated using the agar well diffusion method by assessing the formation of inhibition zones against *S*. *aureus* [31]. *Staphylococcus aureus* cultures were grown overnight in Luria–Bertani (LB) medium at 37°C. The bacterial suspension was then adjusted to approximately 10^8^ CFU/mL. A volume of 100 *μ*L of this suspension was uniformly spread over the surface of LB agar plates using sterilized glass plating beads (4 mm). Wells of 7.5 mm diameter were created in the agar using sterile pipette tips. For the screening of the 17 clinical *S. aureus* isolates, each well was filled with 100 *μ*L of MTE, with sterile deionized water serving as the negative control. For the evaluation of purified MT‐AgNPs against eight *S. aureus* strains, the nanoparticles were dispersed in 15% dimethyl sulfoxide (DMSO), and 100 *μ*L of 15% DMSO was used as the negative control to ensure that the solvent had no inhibitory effect on bacterial growth. Gentamicin (1 *μ*g/mL) was used as a positive control to evaluate antimicrobial activity against *S. aureus* clinical strains isolated from AD patients, based on previously reported minimum inhibitory concentration (MIC) values [32]. The plates were incubated at 37°C for 24–48 h, after which the inhibition zones were measured to assess antibacterial activity.

### 2.4. MIC Test

The MIC of each sample was determined using the broth microdilution method. *Staphylococcus aureus* was cultured overnight at 37°C [33], and the fresh culture was adjusted to ~10^6^ CFU/mL. The bacterial suspension was inoculated into 90 *μ*L of LB medium containing serial dilutions of MTE, in a total volume of 200 *μ*L per well in a 96‐well plate. Plates were incubated at 37°C for 24 h. Bacterial growth was evaluated by measuring optical density at 600 nm (OD_600_) using a microplate spectrophotometer (Thermo Scientific Multiskan GO). MIC was defined as the lowest concentration of the sample that inhibited visible bacterial growth, indicated by no significant increase in OD [34].

### 2.5. Assessment of Biofilm Inhibitory Activity

The MBIC of MTE against *S. aureus* was determined using a crystal violet staining assay, following previously described protocols with slight modifications [35, 36]. Overnight cultures of *S. aureus* were refreshed in LB medium, adjusted to ~10^6^ CFU/mL, and inoculated into 90 *μ*L of LB medium containing serial dilutions of MTE, resulting in a final volume of 200 *μ*L per well in a 96‐well plate. After 24 h of incubation at 37°C, bacterial growth was measured at OD_600_. To assess biofilm formation, the medium was gently removed, and wells were washed three times with sterile deionized water. The plates were air‐dried and stained with 200 *μ*L of 1% (*w*/*v*) crystal violet solution for 30 min at room temperature. Excess stain was discarded, and wells were rinsed three times with sterile deionized water and air‐dried again. To solubilize the bound dye, 200 *μ*L of 30% (*v*/*v*) acetic acid was added to each well and incubated for 10 min at room temperature. Absorbance was measured at 550 nm using a microplate spectrophotometer. Regarding the biofilm inhibitory activity assays, the negative controls were specifically assigned based on the solvent used for each treatment. For the evaluation of MTE against clinical *S. aureus* strains, sterile deionized water was employed as the negative control. In contrast, for the biofilm inhibitory experiments involving MT‐AgNPs, 15% (*v*/*v*) DMSO was used as the negative control to ensure that the observed antibiofilm activity was not influenced by the solvent. In all cases, the untreated bacterial suspension served as the baseline for calculating the percentage of biofilm inhibition. The percentage of biofilm inhibition was calculated using the following equation: biofilm inhibition (*%*) = [(OD_control_ − OD_treatment_)/OD_control_] × 100.

### 2.6. Phytochemical Analysis

The total polyphenol content (TPC) of MTE was determined using a modified Folin–Ciocalteu method [37]. Briefly, 10‐fold diluted MTE was centrifuged at 13,000 rpm for 1 min, and 250 *μ*L of the resulting supernatant was mixed with 125 *μ*L of 50% (*v*/*v*) Folin–Ciocalteu phenol reagent and 250 *μ*L of 95% (*v*/*v*) ethanol. After vortexing, the mixture was incubated in the dark for 5 min, followed by the addition of 250 *μ*L of 5% (*w*/*v*) sodium carbonate solution. The reaction mixture was kept in the dark for 1 h and then centrifuged again at 13,000 rpm for 1 min. Absorbance was measured at 725 nm using a microplate spectrophotometer. Gallic acid solutions ranging from 4 to 500 mg/L were utilized to establish a standard calibration curve [38]. The curve, which exhibited a correlation coefficient (*r* ^2^) greater than 0.95, was employed to determine the TPC, expressed in terms of gallic acid equivalent (GAE).

The total flavonoid content (TFC) of MTE was assessed following the method described in Mahboubi et al.′s [39] study. MTE was centrifuged at 13,000 rpm for 1 min, and 250 *μ*L of supernatant was aliquoted into a reaction tube. Subsequently, 1.5 mL of 95% (*v*/*v*) ethanol, 100 *μ*L of 10% (*w*/*v*) aluminum chloride solution, 100 *μ*L of 1 M potassium acetate, and 2.8 mL of sterile water were added. The mixture was vortexed and incubated in the dark for 40 min. After centrifugation at 13,000 rpm for 1 min, absorbance was measured at 415 nm using a microplate spectrophotometer. Quercetin dilutions ranging from 20 to 2500 mg/L were used to generate a standard calibration curve following the same procedure. The resulting curve was used to calculate the TFC, expressed as quercetin equivalent (QE).

### 2.7. Antioxidant Activity of MTE

The antioxidant activity of MTE was evaluated using two standard methods: the ferric reducing antioxidant power (FRAP) assay and the 1,1‐diphenyl‐2‐picrylhydrazyl (DPPH) radical scavenging assay. For the FRAP assay, a modified protocol based on Saeio et al. [40] was used. Three stock solutions were prepared: (A) 0.3 M sodium acetate buffer (pH 3.6), (B) 10 mM TPTZ (2,4,6‐tripyridyl‐s‐triazine) in 40 mM HCl, and (C) 20 mM FeCl_3_. The FRAP working solution was freshly prepared by mixing the stock solutions in a 10:1:1 (*v*/*v*/*v*) ratio. A total of 100 *μ*L of MTE supernatant was mixed with 700 *μ*L of FRAP working solution, vortexed, and incubated in the dark for 1 h. After centrifugation at 13,000 rpm for 1 min, the absorbance was measured at 593 nm. A standard curve was constructed using FeSO_4_ solutions ranging from 10 to 600 mg/L.

For the DPPH assay [41], 250 *μ*L of MTE supernatant was mixed with 500 *μ*L of 0.2 mM DPPH in methanol. The mixture was vortexed and incubated in the dark at room temperature for 30 min, followed by centrifugation at 13,000 rpm for 1 min. The absorbance of the supernatant (*A*_1_) was measured at 517 nm. A negative control containing only methanol (*A*_2_) was processed in parallel. The DPPH radical scavenging activity was calculated using the equation: DPPH radical scavenging activity (*%*) = [(*A*_2_−*A*_1_)/*A*_2_] × 100.

### 2.8. Tyrosinase Inhibition Assay

The tyrosinase inhibitory activity of test samples was assessed using an L‐3,4‐dihydroxyphenylalanine (L‐DOPA) oxidation–based assay, as previously described with minor modifications [42]. The reactions were conducted in 96‐well microplates, each well containing a total volume of 200 *μ*L: 120 *μ*L of 5 mM L‐DOPA (prepared in 67 mM phosphate buffer, pH 6.8), 40 *μ*L of serially diluted test samples, and two units of tyrosinase. Four groups were established: (A) complete reaction mixture without test samples as control, (B) substrate blank containing L‐DOPA without test samples and tyrosinase, (C) test samples with tyrosinase, and (D) sample blanks containing test samples without tyrosinase. Following incubation at 37°C for 20 min, the enzymatic conversion of L‐DOPA was quantified by measuring absorbance at 490 nm using a microplate spectrophotometer. The percentage of tyrosinase inhibition was calculated using the formula: *%*inhibition = [(*A* − *B*) − (*C* − *D*)]/(*A* − *B*) × 100.

### 2.9. Synthesis of AgNPs Using MTE

To optimize the synthesis of *M. tanarius*–mediated silver nanoparticles (MT‐AgNPs), a single‐factor experimental design was employed to investigate the effects of key synthesis parameters, including reaction temperature (60°C, 70°C, 80°C, 90°C, and 100°C), precursor concentration (1, 3, and 5 mM AgNO_3_), volume ratio of plant extract to silver nitrate solution (1:9, 3:7, and 5:5), and reaction time (15, 30, 45, 60, 75, and 90 min). For each condition, the reaction mixture was maintained at 60°C (except during temperature optimization), stirred at 200 rpm for 1 h, and the formation of MT‐AgNPs was monitored using a microplate spectrophotometer within the wavelength range of 300–700 nm. Optimal conditions for each parameter were selected based on the resulting surface plasmon resonance (SPR) peaks. Following optimization, MT‐AgNPs were synthesized by mixing MTE with a 1 mM aqueous AgNO_3_ solution in a 1:9 (*v*/*v*) ratio. The mixture was heated to 60°C and stirred continuously at 200 rpm for 1 h using a magnetic hot plate stirrer. The initial indication of nanoparticle formation was a visible color change from yellow to brown. The synthesized MT‐AgNPs were further confirmed via SPR analysis using the aforementioned spectrophotometer. Purification of the nanoparticles was performed by centrifugation to remove unreacted plant extract, followed by resuspension in DMSO and a second centrifugation to eliminate residual impurities.

### 2.10. Transmission Electron Microscopy (TEM) Analysis

TEM was used to examine the morphology of the synthesized AgNPs. Purified nanoparticles (3‐4 mg) were resuspended in 10% DMSO, diluted 1:100 in Milli‐Q water, and 10 μL was deposited on Formvar‐ and carbon‐coated 300‐mesh copper grids for 1 min. Grids were examined with a Hitachi H‐7500 microscope (Tokyo, Japan) at 80 kV, and images were captured with an AMT NanoSprint12 CMOS camera.

### 2.11. Fourier‐Transform Infrared (FTIR) Spectroscopy

FTIR spectroscopy was employed to identify functional groups present in the samples by inducing vibrational transitions through interaction with an infrared (IR) beam, resulting in distinct spectral signals. Transmission spectra of the purified AgNPs were recorded using a FTIR instrument (ALPHA II Compact FTIR Spectrometer, Bruker, Billerica, Massachusetts). For comparative analysis, spectra of MTE and AgNO_3_ were also obtained.

### 2.12. X‐Ray Diffraction (XRD) Analysis

To obtain powdered AgNPs, the colloidal solution underwent centrifugation at 10,000 rpm for 30 min at room temperature. The resulting pellet was collected, air‐dried, and subsequently placed into the sample holder for analysis. XRD was conducted using a SHIMADZU XRD‐6000 diffractometer with FeK*α* radiation (*λ* = 1.9372 Å), operating at 30 kV and 25 mA. Scanning occurred over a 2*θ* range of 15°–85° with a 2‐s time constant. XRD patterns were initially processed using the instrument′s proprietary software, and the resulting data were subsequently plotted using Origin (OriginLab, United States) to generate the final diffraction profiles for presentation. The average crystallite size of the AgNPs was determined using the Debye–Scherrer equation: crystalline size (*D*) = *k* *λ*/*β* cos *θ*, where *D* represents the average crystallite size (in nm), *k* is the shape factor (0.94), *λ* is the X‐ray wavelength (1.5406 Å), *β* is the full width at half maximum (FWHM) of the most intense diffraction peak (in radians), and *θ* is the Bragg angle. This analysis provided significant insights into the crystalline nature and structural properties of the synthesized AgNPs.

### 2.13. Dye Degradation Assay

The catalytic activity of MT‐AgNPs was evaluated using Congo red as a model dye, following a method adapted from Indana et al. [43]. In addition to assessing the catalytic performance of MT‐AgNPs, sodium borohydride (NaBH_4_) was included in the reaction mixture. This compound serves to enhance the reduction capabilities of the nanoparticles. The reaction mixture consisted of 100 *μ*L of MT‐AgNP suspension in DMSO, 750 *μ*L of 3% (*w*/*v*) Congo red solution, and 150 *μ*L of 0.1 M NaBH_4_. Four control groups were prepared: (a) 100 *μ*L of DMSO with dye and NaBH_4_, (b) 100 *μ*L of distilled water with dye and NaBH_4_, (c) 100 *μ*L of MT‐AgNPs with dye and distilled water, and (d) 100 *μ*L of DMSO with dye and distilled water. Each mixture was exposed to halogen light under continuous stirring, and the degradation of Congo red was monitored at 10‐min intervals using a microplate spectrophotometer until complete decolorization was achieved.

### 2.14. Statistical Analysis

For comparisons between two groups, statistical analysis was performed using an unpaired Student′s *t*‐test. Asterisks in the figures indicate levels of significance as follows:  ^∗^ *p* < 0.05 and  ^∗∗^ *p* < 0.01. For comparisons involving more than two groups, one‐way analysis of variance (ANOVA) followed by Tukey′s post hoc test was used. Statistical significance was defined as *p* < 0.05.

## 3. Results

### 3.1. Isolation, Identification, and Broad‐Spectrum Inhibitory Effect of MTE on Clinical *S. aureus* Strains From AD Patients

Following isolation and culturing on MSA plates, a total of 27 bacterial strains were obtained from samples collected from 16 AD patients. Species identification was performed via PCR amplification, followed by RAPD analysis to exclude identical strains. BLAST analysis against the NCBI nucleotide database confirmed that 17 of the isolates were identified as *S. aureus*. Among these, strains SA12 and SA13 tested positive for the *mecA* gene, suggesting the potential presence of MRSA.

The antibacterial activity of MTE against all 17 clinical *S. aureus* strains was assessed using the agar well diffusion assay. Sterile water was used as a negative control. All tested strains showed clear inhibition zones surrounding the wells containing MTE, whereas no inhibition was observed in the control wells (Figure S1). These results demonstrate that MTE consistently inhibited the growth of all clinical *S. aureus* isolates tested, including potential MRSA strains.

### 3.2. MIC Determination and Biofilm Inhibition of MTE Against Clinical *S. aureus* Strains

To evaluate the antibacterial potential of MTE for possible clinical applications, a subset of clinical *S. aureus* strains was selected based on biofilm‐forming activity. Strains SA91, SA92, and SA81 were selected, with SA91 exhibiting relatively weak biofilm formation, while SA92 and SA81 were the strongest biofilm producers among the purified strains. Strains SA12 and SA13, which tested positive for the *mecA* gene and, thus, were identified as potential MRSA, were also included due to their notable ability to form biofilms. Two additional strains, SA7 and SA11, which showed no detectable biofilm formation, were randomly selected to increase strain diversity. All assays were performed using MTE prepared by boiling 10 g of fresh *M. tanarius* leaves in 100 mL of sterile deionized water for 15 min, as described in the Materials and Methods section.

MIC values were defined based on spectrophotometric measurements of bacterial turbidity after 24 h of incubation (Table 1). Complete growth inhibition was observed at an MTE concentration of 300 *μ*L/mL for strains SA7, SA11, SA13, SA81, and SA92. Interestingly, strain SA12 showed greater sensitivity, with inhibition observed at 200 *μ*L/mL, while SA91 required a higher concentration of 400 *μ*L/mL for complete inhibition, indicating variability in MTE susceptibility among the clinical strains.

**Table 1:** Minimum inhibitory concentration (MIC) values of *Macaranga tanarius* extract (MTE) and *M. tanarius*–mediated silver nanoparticles (MT‐AgNPs) against *Staphylococcus aureus* isolates.

Strains tested	SA7	SA11	SA12	SA13	SA81	SA91	SA92	Unit
MTE	300	300	200	300	300	400	300	*μ*L/mL
MT‐AgNPs	18.75	18.75	9.375	9.375	9.375	9.375	9.375	*μ*g/mL

**Figure 1:**
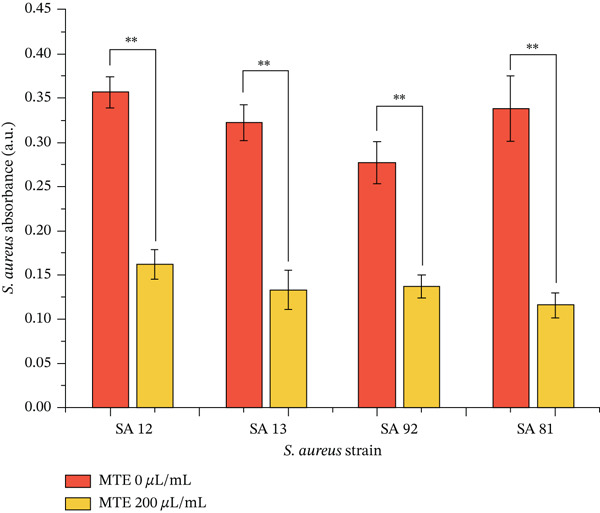
Inhibitory effect of *Macaranga tanarius* extract (MTE) on biofilm formation by *Staphylococcus aureus*. Biofilm formation by different *S. aureus* strains (SA12, SA13, SA92, and SA81) was quantified using crystal violet staining, and the optical density (OD) at 550 nm was measured with a microplate spectrophotometer. Treatment with 200 *μ*L/mL MTE significantly reduced biofilm formation compared to untreated controls (red bar: control; yellow bar: MTE‐treated). The inhibition rates were 53.5*%* ± 8.3*%* for SA12, 60.4*%* ± 6.6*%* for SA13, 50.4*%* ± 4.6*%* for SA92, and 64.3*%* ± 4.4*%* for SA81 (mean ± SD). Statistical significance was determined using a *t*‐test, with  ^∗∗^ *p* < 0.01.

To evaluate the inhibitory effect of MTE on biofilm formation, bacterial cultures were treated with a subinhibitory concentration of MTE (200 *μ*L/mL), selected based on MIC results. This concentration was used to evaluate interference with the initial formation of biofilms rather than disruption of preformed biofilms. The inhibition ability of MTE against biofilm formation of different *S. aureus* strains was as follows: SA12, 53.5*%* ± 8.3*%*; SA13, 60.4*%* ± 6.6*%*; SA92, 50.4*%* ± 4.6*%*; and SA81, 64.3*%* ± 4.4*%* (mean ± standard deviation) (Figure 1). Strain SA91 exhibited weak biofilm formation and thus was excluded from this assay.

### 3.3. Phytochemical, Antioxidant, and Tyrosinase Inhibition Activities of MTE

MTE contains a variety of bioactive compounds, among which polyphenols and flavonoids were quantitatively measured. The TPC was measured at 2429 ± 33.7 mg GAE/100 mL, while the TFC was 236.3 ± 10.7 mg QE/100 mL.

The antioxidant activity of MTE was assessed using two standard assays. In the DPPH radical scavenging assay, MTE demonstrated 82.72*%* ± 3.12*%* activity, while in the FRAP assay, the reducing capacity was measured at 20.36 ± 0.02 mg FeSO_4_/100 mL. These results highlight the strong free radical scavenging and reducing capacities of MTE, indicating its potential to mitigate oxidative stress. Additionally, tyrosinase catalyzes the oxidation of L‐DOPA, leading to the production of melanin, which is involved in hyperpigmentation‐related skin disorders such as dullness and freckles. Therefore, inhibiting tyrosinase activity is important in both medical and cosmetic applications. Tyrosinase inhibition activity of MTE was evaluated over a 2‐h period, with measurements recorded at 30‐min intervals. The inhibition percentages recorded were approximately 44.5*%* ± 0.8*%*, 45.3*%* ± 3.3*%*, 43.5*%* ± 0.3*%*, and 44.9*%* ± 3.8*%*, respectively. Corresponding to these time points, the kojic acid equivalents (mg/g) were 6.07 ± 0.2, 8.5 ± 0.3, 6.6 ± 0.4, and 6.0 ± 0.2 (Table 2).

**Table 2:** Bioactive properties of *Macaranga tanarius* extract (MTE): Polyphenol and flavonoid content, antioxidant activity, and tyrosinase inhibition. GAE, gallic acid equivalent; QE, quercetin equivalent; KAE, kojic acid equivalent. Each assay was performed in triplicate, and the data are presented as mean ± SD.

Bioactive properties of *Macaranga tanarius* extract (MTE)							
Total polyphenol content (mg GAE/100 mL)							
2429 ± 33.7							
Total flavonoid content (mg QE/100 mL)							
236.3 ± 10.7							
FRAP (mg FeSO_4_/100 g)							
20.36 ± 0.02							
DPPH scavenging (%)							
82.72 ± 3.12							
Tyrosinase inhibition activity							
Inhibition percentage (%)				KAE (mg/g)			
0.5 h	1 h	1.5 h	2 h	0.5 h	1 h	1.5 h	2 h
44.5 ± 0.8	45.3 ± 3.3	43.5 ± 0.3	44.9 ± 3.8	6.07 ± 0.2	8.5 ± 0.3	6.6 ± 0.4	6 ± 0.2

### 3.4. Optimization of Green Synthesis Conditions for MT‐AgNPs

**Figure 2:**
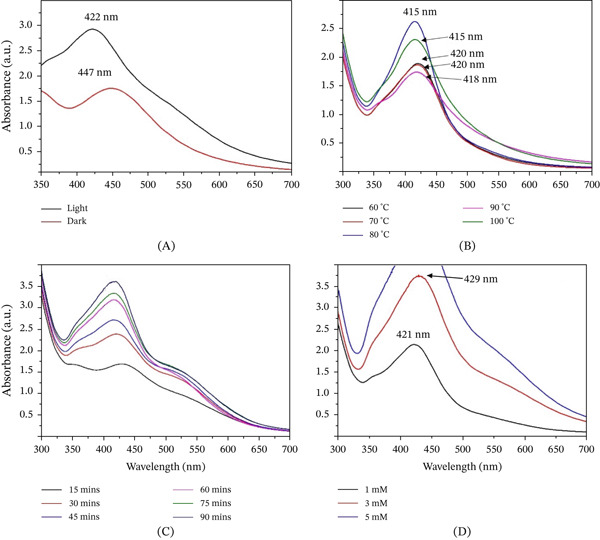
Optimization of green synthesis conditions for *Macaranga tanarius*–synthesized silver nanoparticles (MT‐AgNPs). (A) Surface plasmon resonance (SPR) analysis revealed reduced AgNP formation under dark conditions, as indicated by a redshift from 422 to 447 nm and decreased absorbance. (B) Temperature‐dependent synthesis (60°C–100°C) showed maximal SPR intensity at 80°C, with peak positions between 415 and 420 nm corresponding to particle sizes of ~10–40 nm. (C) Increasing synthesis time (15–90 min) led to higher SPR absorbance and peak shifts between 415 and 430 nm, indicating enhanced nanoparticle formation. (D) Varying AgNO_3_ concentrations (1–5 mM) resulted in SPR peaks from 421 to 429 nm, with higher precursor levels increasing nanoparticle yield but also shifting peak positions, suggesting larger particle sizes. Optimal synthesis was achieved under light exposure with 1 mM AgNO_3_ at 80°C for 90 min.

The synthesis of MT‐AgNPs was optimized by evaluating the influence of light exposure, temperature, reaction time, and silver nitrate concentration on nanoparticle formation. This process relied on the reduction of AgNO_3_ by biomolecules, resulting in the formation of capped and stabilized AgNPs. Under dark conditions, the SPR peak shifted from 422 to 447 nm, with a corresponding decrease in peak intensity from 3 to 1.75 a.u. (Figure 2A), indicating a reduction in AgNP formation in the absence of light. Temperature‐dependent synthesis was examined across a range of 60°C–100°C in 10°C increments (Figure 2B). The SPR peaks ranged between 415 and 420 nm, suggesting that the AgNPs formed were approximately 10–40 nm in size. The SPR intensity increased with temperature, peaking at 80°C and declining at higher temperatures. The influence of reaction time was assessed, ranging from 15 to 90 min (Figure 2C). The SPR peaks observed between 415 and 430 nm indicated an increase in absorbance as synthesis time progressed, suggesting enhanced AgNP formation with longer reaction times. AgNO_3_ concentrations of 1, 3, and 5 mM were also tested (Figure 2D). SPR peaks ranged from 421 to 429 nm, corresponding to AgNP sizes of approximately 20–50 nm. Higher precursor concentrations led to increased absorbance values, reflecting greater AgNP yield. However, the peak shift from 421 to 429 nm suggested that higher precursor concentrations produced larger nanoparticles. Based on these observations, the optimal synthesis conditions selected for subsequent experiments were light exposure, 1 mM AgNO_3_, a reaction temperature of 80°C, and a synthesis time of 90 min.

### 3.5. Characterization of AgNPs

**Figure 3:**
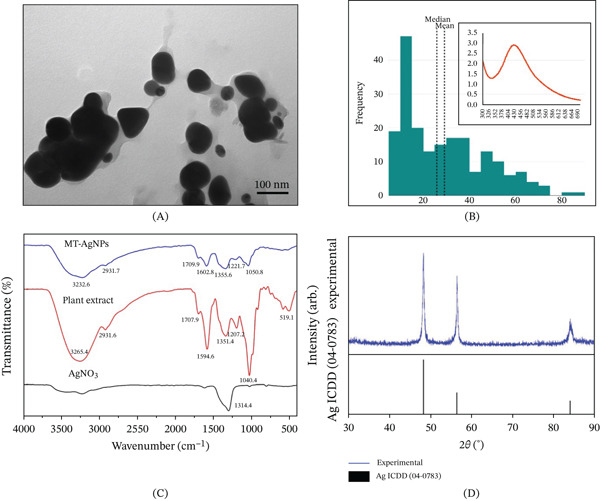
Characterization of *Macaranga tanarius*–synthesized silver nanoparticles (MT‐AgNPs). (A, B) Transmission electron microscopy (TEM) images show predominantly spherical nanoparticles with a size distribution indicating that approximately 80% of particles fall within 10–56 nm. (B) The UV–visible spectrum displays a sharp surface plasmon resonance (SPR) peak at ~430 nm, characteristic of well‐dispersed spherical AgNPs. (C) Fourier‐transform infrared (FTIR) spectra of MTE and MT‐AgNPs reveal similar functional groups, indicating the presence of bioactive compounds capping the nanoparticles. (D) X‐ray diffraction (XRD) analysis confirms the crystalline nature of the synthesized AgNPs, with diffraction peaks corresponding to characteristic crystallographic planes of metallic silver.

TEM confirmed the successful synthesis of AgNPs. TEM images revealed that the nanoparticles were predominantly spherical (Figure 3A), with sizes ranging from approximately 6 to 87 nm. The mean particle size was 29.2 nm, and the median was 25.88 nm, indicating a right‐skewed distribution. Most nanoparticles were distributed within the 10–56 nm range, encompassing approximately 80% of the total population (Figure 3B). The UV–visible spectrum of the purified MT‐AgNPs exhibited a distinct SPR peak at approximately 430 nm (Figure 3B), which is characteristic of well‐dispersed spherical AgNPs. This absorption peak lies within the typical SPR range (390–450 nm) reported for biogenically synthesized AgNPs in the 20–60 nm size range. The presence of a sharp, symmetrical peak indicates successful synthesis and purification of stable AgNPs with minimal aggregation. Similar spectral features have been widely reported in plant‐mediated AgNP synthesis, supporting the formation of metallic AgNPs capped with bioactive compounds.

FTIR spectroscopy was used to identify functional groups present in MTE and to characterize the surface chemistry of MT‐AgNPs (Figure 3C). MTE exhibited absorption peaks at 3265, 2931, 1707, 1594, 1351, 1207, 1040, and 519 cm^−1^, while MT‐AgNPs showed peaks at 3232, 2931, 1709, 1602, 1355, 1221, and 1054 cm^−1^. The corresponding peak assignments are summarized in Table 3. The broad absorption bands at 3265.4 cm^−1^ (MTE) and 3232.6 cm^−1^ (MT‐AgNPs) are attributed to the hydroxyl (‐OH) stretching vibrations. The small absorption peaks at 2931.6 cm^−1^ (MTE) and 2931.7 cm^−1^ (MT‐AgNPs) correspond to C‐H stretching vibrations in methyl and methylene groups. The bands at 1707.9 cm^−1^ (MTE) and 1709 cm^−1^ (MT‐AgNPs) are assigned to C=O stretching vibrations of carbonyl groups. Peaks at 1594.6 cm^−1^ (MTE) and 1602.8 cm^−1^ (MT‐AgNPs) correspond to C=C stretching vibrations in the aromatic groups of benzene rings. The peaks at 1351.4 cm^−1^ (MTE) and 1355.6 cm^−1^ (MT‐AgNPs) are also associated with C=O stretching vibrations, while the smaller peaks at 1207.2 cm^−1^ (MTE) and 1221.7 cm^−1^ (MT‐AgNPs) indicate C‐H (CH_3_) group vibrations. The absorption peaks at 1040.4 cm^−1^ (MTE) and 1054.8 cm^−1^ (MT‐AgNPs) correspond to C‐O stretching vibrations in primary and secondary alcohols, and the small peak at 519.1 cm^−1^ is attributed to the aromatic bending vibrations of the C‐H group.

**Table 3:** Characteristic FTIR band assignments for *Macaranga tanarius* extract (MTE) and *M. tanarius*–mediated silver nanoparticles (MT‐AgNPs).

No.	FTIR peak (cm^−1^)		Assignment
	Plant extract	MT‐AgNps	
1	3265.4	3302.5	‐OH stretching vibration
2	2931.6	2933.7	C‐H stretching vibration [75]
3	1707.9	1712.0	Stretching vibration of C=O containing functional groups (e.g., carbonyl, acid, and ester) [76]
4	1594.6	1602.8	C=C stretching vibration in aromatic groups of benzene ring [76]
5	1351.4	1349.4	C=O stretching vibration
6	1207.2	1215.5	C‐H (CH_3_) bending symmetric
7	1040.4	1048.6	C‐O stretching vibration for alcohol, phenol
8	519.1		C‐O bond

The crystalline structure of the synthesized AgNPs was confirmed by XRD analysis. The XRD pattern exhibited distinct diffraction peaks at approximately 2*θ* = 48^°^, 57°, and 84°, corresponding to the (200), (311)/(220), and (222) planes of the face‐centered cubic (fcc) structure of metallic silver, as referenced by JCPDS Card No. 04‐0783. These characteristic peaks confirm the formation of highly crystalline AgNPs. The absence of additional diffraction peaks associated with impurities suggests that the synthesized nanoparticles possess high purity and well‐defined phase identity. Moreover, the observed diffraction pattern closely matches the standard ICDD reference (Figure 3D). The average crystallite size of the AgNPs was calculated to be 32.16 nm using the Debye–Scherrer equation, based on the FWHM of the most intense peak at 2*θ* = 48^°^, further confirming the crystalline nature of the particles.

### 3.6. Evaluation of Antibacterial Efficacy and Biofilm Inhibition of MT‐AgNPs

**Figure 4:**
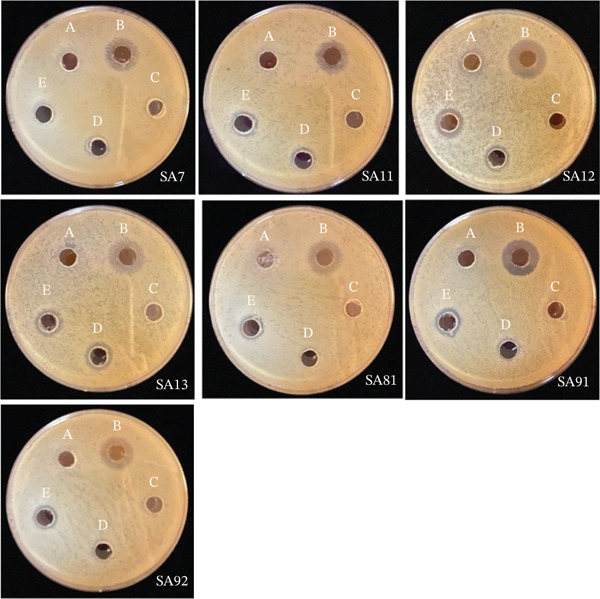
Antibacterial activity of purified *Macaranga tanarius*–synthesized silver nanoparticles (MT‐AgNPs) against eight *Staphylococcus aureus* strains. (A) DMSO negative control and (C) water negative control showed no inhibition zones, confirming no antibacterial effect. (B) MT‐AgNPs dissolved in DMSO produced clear inhibition zones against all tested strains, including potential MRSA, demonstrating strong antibacterial activity. (D) Gentamicin (1 *μ*g/mL) served as a positive control to validate the assay. (E) MT‐AgNPs dissolved in water also exhibited antibacterial activity but with smaller inhibition zones compared to those dissolved in DMSO.

In the MT‐AgNP agar well diffusion assay, no inhibition zones were observed in water or DMSO negative control groups, confirming that they did not contribute to antibacterial activity. In contrast, treatment with 1 mg/mL MT‐AgNPs resulted in clear inhibition zones against all eight tested *S. aureus* strains, including potential MRSA (Figure 4). Notably, purified MT‐AgNPs dissolved in DMSO produced larger inhibition zones compared to those dissolved in water. Consequently, MT‐AgNPs dissolved in DMSO were selected for subsequent experiments. Gentamicin was used as a positive control and confirmed the proper execution of the experiment.

**Figure 5:**
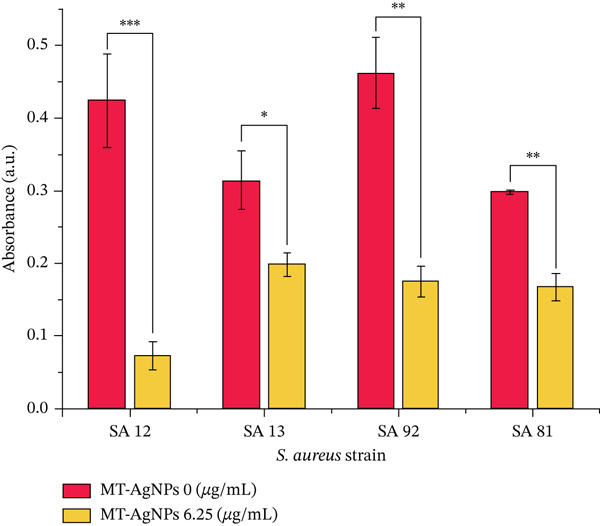
Biofilm inhibition activity of *Macaranga tanarius*–synthesized silver nanoparticles (MT‐AgNPs) against *Staphylococcus aureus*. The red bar indicates untreated controls, while the yellow bar represents biofilms treated with 6.25 *μ*g/mL MT‐AgNPs. At this concentration (two‐thirds of the MIC), MT‐AgNPs significantly inhibited biofilm formation across all tested strains. The average biofilm formation percentages were 82.8*%* ± 4.5*%* (SA12), 36.9*%* ± 5.1*%* (SA13), 62.0*%* ± 4.5*%* (SA92), and 43.8*%* ± 6.0*%* (SA81). Statistical significance was determined by *t*‐test, with  ^∗^ *p* < 0.05,  ^∗∗^ *p* < 0.01, and  ^∗∗∗^ *p* < 0.001.

MIC results further demonstrated that MT‐AgNPs effectively inhibited the growth of all tested *S. aureus* strains. Strains SA7 and SA11 exhibited MIC values of 18.75 *μ*g/mL, while lower MICs of 9.375 *μ*g/mL were observed for strains SA12, SA13, SA81, SA91, and SA92, including potential MRSA strains (Table 1). In addition to their antibacterial activity, MT‐AgNPs also showed biofilm inhibition capabilities. When applied at two‐thirds of the MIC (6.25 *μ*g/mL), MT‐AgNPs significantly inhibited biofilm formation compared to the untreated control group (Figure 5). The average biofilm formation percentages for SA12, SA13, SA92, and SA81 were 82.8*%* ± 4.5*%*, 36.9*%* ± 5.1*%*, 62.0*%* ± 4.5*%*, and 43.8*%* ± 6.0*%*, respectively. These findings suggest that MT‐AgNPs not only suppress planktonic bacterial growth but also reduce biofilm development in clinically relevant *S. aureus* strains.

### 3.7. Degradation of Congo Red Facilitated by MT‐AgNPs

To verify that the degradation of Congo red was driven by the catalytic reduction of MT‐AgNPs, four control groups were examined alongside the experimental group. As shown in Figure S2A, no significant changes in the absorbance spectra were observed in any of the control groups, which included reaction mixtures lacking MT‐AgNPs (DMSO or water with dye and NaBH_4_), mixtures lacking the reducing agent (MT‐AgNPs with dye and water), and solvent‐only controls (DMSO with dye). The persistence of the characteristic absorption peak at approximately 500 nm in these controls indicates that neither NaBH_4_ alone nor MT‐AgNPs alone could induce dye degradation under the tested conditions. In distinct contrast, the complete reaction system containing both MT‐AgNPs and NaBH_4_ exhibited a rapid and marked decrease in absorbance over time (Figure S2B). The absorbance dropped from an initial value of > 3.0 a.u. to lower levels within 50 min, demonstrating the high catalytic efficiency of the synthesized nanoparticles.

## 4. Discussion

The present study demonstrates, for the first time, the successful green synthesis of AgNPs using MTE and highlights their promising antibacterial and antibiofilm activities against clinical *S. aureus* strains isolated from AD patients. The strong inhibitory effects of both MTE and the biogenically synthesized MT‐AgNPs against a wide range of *S. aureus* strains—including potential MRSA—underscore the therapeutic potential of this plant‐mediated nanotechnology platform. The observed efficacy in reducing biofilm formation, together with the high polyphenol and flavonoid content of MTE, suggests a synergistic contribution of phytochemicals to bacterial suppression. Additionally, the notable antioxidant and tyrosinase inhibitory activities may contribute to nanoparticle stability. These findings position MTE as an effective antistaphylococcal agent that also serves as a sustainable, bioactive reducing precursor for the eco‐friendly production of AgNPs. These MT‐AgNPs are specifically designed to target *S. aureus* colonization and associated complications in AD skin.

### 4.1. Therapeutic Potential of *M. tanarius*–Derived AgNPs

The present study demonstrates that the aqueous extract of *M. tanarius* leaves exhibits significant antibacterial activity against multiple clinical *S. aureus* strains isolated from the skin of patients with AD. Notably, this includes two strains harboring the *mecA* gene, suggesting potential MRSA phenotypes. These findings are particularly relevant given the increasing prevalence of antibiotic‐resistant *S. aureus* in AD patients and its association with disease exacerbation and impaired skin barrier function [46]. The ability of the extract to inhibit not only planktonic bacterial growth but also biofilm formation is of particular interest, as biofilms contribute to chronic infection, antibiotic resistance, and treatment failure [47].

Previous studies have reported that phenolic compounds, flavonoids, and other plant‐derived secondary metabolites can interfere with bacterial quorum sensing and biofilm development [48]. Phytochemical constituents previously identified in *Macaranga* plants, such as prenylated flavonoids, may underlie the observed antimicrobial and antibiofilm effects [49–52]. Additionally, the aqueous extract of *M. tanarius* leaves contains notable concentrations of bioactive compounds, with polyphenols measured at 2429 ± 33.7 mg GAE/100 g and flavonoids at 236.3 ± 10.7 mg QE/100 mL. These values are relatively high compared to other plant extracts commonly reported in the literature [53, 54]. The high concentrations of polyphenols and flavonoids in MTE likely contribute to its observed antimicrobial and antibiofilm properties. Polyphenols are recognized for their antioxidant, antimicrobial, and anti‐inflammatory properties, while flavonoids have been shown to disrupt bacterial cell membranes and modulate quorum sensing, thus inhibiting biofilm formation [55]. Given the high levels of these bioactive compounds, it is reasonable to conclude that they play a critical role in the effectiveness of the extract against *S. aureus*, particularly in MRSA strains resistant to conventional antibiotics. Although the present study did not directly measure reactive oxygen species (ROS) generation or membrane disruption, previous studies have shown that AgNPs can exert antibacterial effects through multiple mechanisms. These mechanisms include the induction of oxidative stress and damage to bacterial membranes [56, 57]. The phytochemicals present in *M*. *tanarius*–mediated AgNPs may further enhance these effects by facilitating nanoparticle–bacteria interactions or stabilizing reactive species. Based on these considerations, it is plausible that the observed inhibition of AD‐associated *S*. *aureus* strains involves similar oxidative and membrane‐disruptive mechanisms. Future investigations using ROS assays, membrane integrity tests, and molecular analyses will help to clarify the precise pathways underlying the antimicrobial and antibiofilm activities of these biosynthesized nanoparticles. Together, these findings highlight the potential of *M. tanarius* leaf extract as a promising candidate for alternative or adjunctive therapy targeting *S. aureus*, especially in the context of AD‐associated infections and MRSA‐related complications [58–60].

The observed tyrosinase inhibitory activity of the *M. tanarius* aqueous extract highlights its potential as a natural depigmenting agent. Given the increasing demand for safer, plant‐derived alternatives to synthetic skin‐whitening compounds such as hydroquinone and kojic acid, MTE presents a promising avenue. Its potential applications span cosmetic and dermatological fields [61]. Furthermore, the use of water as the extraction solvent not only aligns with principles of green chemistry but also enhances the extract′s compatibility for formulation in topical products. Future studies focusing on the isolation of active constituents and in vivo efficacy evaluations will be essential to further validate its potential as a functional ingredient in skin care formulations targeting hyperpigmentation disorders.

### 4.2. FTIR Analysis of Phytochemical Functional Groups Associated With MT‐AgNPs

The FTIR analysis was conducted to evaluate the chemical interactions and functional groups present in both MTE and the synthesized MT‐AgNPs. As illustrated in Figure 3C and detailed in Table 3, both samples exhibited similar absorption patterns. This indicates that the phytochemicals present in the extract participated in the reduction and stabilization processes during nanoparticle synthesis. The broad absorption bands around 3265 cm^−1^ (MTE) and 3232 cm^−1^ (MT‐AgNPs) correspond to O‐H stretching vibrations, characteristic of hydroxyl groups in polyphenols and flavonoids. Peaks at ~1707–1709 cm^−1^ are associated with C=O stretching in carbonyl groups, while bands near ~1594–1602 cm^−1^ correspond to aromatic C=C stretching vibrations. Other notable peaks, including those related to C‐H stretching and C‐O vibrations, further confirm the presence of diverse functional groups derived from plant metabolites. These shared peaks in both the functional group region and fingerprint region suggest that many of the original bioactive constituents in MTE are retained on the surface of MT‐AgNPs. This is significant because flavonoids, stilbenes, and other phenolic compounds are not only responsible for reducing silver ions but also confer biological activity to the nanoparticles. The presence of these phytochemicals on the nanoparticle surface supports a coordinated therapeutic effect, primarily focusing on *S. aureus* inhibition, while secondary antioxidant and anti‐inflammatory properties assist in mitigating AD‐related skin damage [62, 63]. Previous studies have emphasized the role of plant‐derived functional groups in modulating the biological activity of nanoparticles. In this study, the retention of these groups in MT‐AgNPs may account for their potent antibacterial effects, including inhibition of MRSA strains isolated from AD patients. The FTIR findings, therefore, suggest that MT‐AgNPs are capped with phytochemical functional groups, providing a potential dual mechanism of antimicrobial action.

### 4.3. Antibacterial Mechanism of MT‐AgNPs

The potent antibacterial activity of MT‐AgNPs against clinical *S. aureus* isolates, including *mecA*‐positive strains, is driven by a sophisticated multitargeted mechanism. Since the assays were conducted in the absence of light, the inhibition stems from intrinsic chemophysical processes rather than extrinsic photo‐activated pathways. Compared to nanoparticles produced via traditional chemical synthesis, the green‐synthesized MT‐AgNPs exhibit enhanced biological integration and potentially reduced systemic toxicity, which is critical for treating sensitive skin conditions like AD [64].

First, the release of silver ions (Ag^+^) via oxidative dissolution is a dominant factor. In the aqueous environment, MT‐AgNPs undergo controlled oxidation to release Ag^+^ ions, which act as “soft acids” with a high affinity for sulfur‐containing biomolecules. These ions bind to thiol groups (‐SH) in essential respiratory enzymes and transport proteins, irreversibly inactivating them and disrupting cellular metabolism [65]. Second, the nanoparticles exert toxicity through direct physical interaction and membrane disruption. MT‐AgNPs attach to the bacterial cell wall, penetrating the thick peptidoglycan layer of Gram‐positive *S. aureus*. This accumulation causes severe physical destabilization of the membrane and alters the surface charge, leading to the formation of “pits” or structural defects. These changes increase membrane permeability, resulting in the leakage of intracellular contents—such as proteins, K^+^ ions, and reducing sugars—and the dissipation of the proton motive force [66–68].

Furthermore, while extrinsic photocatalytic ROS is precluded in dark incubation, MT‐AgNPs induce significant intrinsic intracellular ROS generation. The interaction of MT‐AgNPs and internalized Ag^+^ with the bacterial electron transport chain (ETC) uncouples aerobic respiration. This triggers the abnormal accumulation of superoxide (·O2^−^) and hydroxyl radicals (·OH) within the cell [69, 70]. This internal oxidative stress triggers lipid peroxidation of the cell membrane, causes the denaturation of ribosomal proteins, and leads to severe fragmentation of genomic DNA, ultimately triggering bacterial cell death [71]. Collectively, these results suggest that MT‐AgNPs target bacteria through a multifaceted mode of action involving sustained ion release, physical structural damage, and irreversible metabolic disruption.

### 4.4. Comparative Analysis of Antibacterial and Antibiofilm Efficacy

The antibacterial efficacy of MT‐AgNPs (MIC: 9.375–18.75 *μ*g/mL) demonstrates superior or highly competitive performance compared to other biogenic AgNPs reported in recent literature. For instance, AgNPs synthesized using *Azadirachta indica* (Neem) leaf extract exhibited a significantly higher MIC of 50 *μ*g/mL against *S. aureus* [1], while those from Aloe vera showed an MIC of 25 *μ*g/mL [2]. Even when compared to AgNPs derived from other medicinal plants like *Syzygium cumini* (MIC: 31.25 *μ*g/mL) [3], our MT‐AgNPs show enhanced potency at lower concentrations. This suggests that the specific prenylated flavonoids and stilbenes present in *M. tanarius* may provide a more biologically active capping layer, facilitating deeper penetration or stronger interaction with the bacterial cell envelope. Regarding biofilm inhibition, MT‐AgNPs achieved significant suppression at a sub‐MIC level of 6.25 *μ*g/mL (inhibiting up to 63%–82% depending on the strain). In comparison, AgNPs synthesized via *Piper longum* required a much higher concentration of 25 *μ*g/mL to achieve a similar 60% reduction in *S. aureus* biofilm [4]. The ability of MT‐AgNPs to disrupt the biofilm matrix at such low dosages is particularly relevant for treating AD, where *S. aureus* often resides in recalcitrant biofilm communities that are notoriously resistant to conventional therapy.

### 4.5. Catalytic Degradation of Congo Red and Its Antibacterial Significance

The catalytic performance of MT‐AgNPs in Congo red degradation highlights their potential as efficient nanocatalysts for environmental remediation (see Figure S2) while also serving as a crucial physicochemical indicator of their surface reactivity and electron‐shuttling capability. This performance is primarily attributed to the high surface area‐to‐volume ratio and the abundance of exposed reactive sites on the AgNPs, which facilitate efficient adsorption of dye molecules and subsequent electron transfer reactions. The mechanism is best described by the electron relay effect, where the MT‐AgNPs act as an intermediate redox catalyst to facilitate the transfer of electrons from the donor (BH_4_^−^) to the acceptor (Congo red) [72]. By accepting electrons from nucleophilic BH_4_^−^ ions and conveying them to the electrophilic dye, the nanoparticles effectively lower the activation energy required for the reaction, allowing it to proceed rapidly at room temperature [73]. Importantly, this same capacity for catalytic electron transfer is fundamental to the antibacterial efficacy of AgNPs; it facilitates the disruption of bacterial respiratory chains and the subsequent induction of intracellular ROS, as previously discussed.

Phytochemicals retained from the *M. tanarius* leaf extract, such as polyphenols and flavonoids, may further enhance this activity. Beyond acting as reducing and stabilizing agents, these organic capping molecules form a layer that not only prevents nanoparticle aggregation but may also facilitate the initial adsorption of dye molecules onto the catalyst surface, thereby increasing the local concentration of the reactant [74]; this capping layer can also participate in catalytic electron shuttling [75]. The observed catalytic enhancement in the presence of NaBH_4_ confirms this synergistic mechanism, accelerating the reductive cleavage of the azo bond (‐N=N‐) and promoting the structural transformation of the dye. In the context of this study′s microbiological focus, the high catalytic efficiency of MT‐AgNPs serves as a nonbiological proxy to validate their potent oxidative potential against clinical *S. aureus* isolates. Overall, these results underscore the potential of green‐synthesized AgNPs as a sustainable solution for treating dye‐contaminated wastewater while simultaneously providing a coherent physicochemical justification for their robust antimicrobial activity [76].

Despite the significant bioactivities demonstrated by MT‐AgNPs, this study has several limitations that should be addressed in future research. First, the clinical isolates were exclusively obtained from volunteers in Japan, which may limit the geographic representativeness of the findings. Since *S. aureus* genetic lineages can vary by region, further validation with globally diverse strains is necessary. Second, the current evaluation relies on in vitro assays; therefore, in vivo toxicity and biocompatibility assessments on human skin models are essential before clinical application. Lastly, while we confirmed the reduction of Ag^+^ to metallic silver, the precise molecular pathways and mechanistic interactions between the *M. tanarius* phytochemicals and the bacterial targets remain to be fully elucidated through transcriptomic or proteomic analyses.

## 5. Conclusion

In conclusion, this study demonstrates the successful green synthesis of AgNPs using *M. tanarius* leaf extract, creating a platform that bridges green chemistry with biomedical and potential agricultural applications. Rather than merely exhibiting multiple bioactivities, the synthesized MT‐AgNPs function as a coordinated therapeutic agent primarily targeted at neutralizing MDR *S. aureus* in AD patients. By prioritizing the antistaphylococcal and antibiofilm efficacy, this work provides a viable plant‐mediated alternative to conventional antibiotics. Furthermore, the integration of these bioactive metabolites suggests a broader ecological and economic role, potentially benefiting sustainable agriculture through eco‐friendly antimicrobial crop protection. While these in vitro results establish a strong foundation, future research focusing on in vivo toxicity and mechanistic pathways will be essential to translate this bio‐inspired nanotechnology into clinical and industrial practice.

## Funding

This study was funded by the Ministry of Science and Technology, Taiwan, 10.13039/501100004663, MOST 111‐2621‐B‐018‐001.

## Conflicts of Interest

The authors declare no conflicts of interest.

## Supporting information


**Supporting Information** Additional supporting information can be found online in the Supporting Information section. Figure S1: Agar well diffusion assay results showing the antibacterial activity of *Macaranga tanarius* extract (MTE) against 17 clinical *Staphylococcus aureus* isolates from atopic dermatitis patients, including mecA‐positive strains. Details are provided in the Supporting Information Data. Figure S2: UV–Vis spectral results showing the catalytic degradation efficiency of *Macaranga tanarius*–synthesized silver nanoparticles (MT‐AgNPs) against Congo red in the presence of NaBH_4_. Details are provided in the Supporting Information Data.

## Data Availability

The data that support the findings of this study are available from the corresponding author upon reasonable request.
