# Supplementary material for: Green Synthesis of Silver Nanoparticles From Macaranga tanarius (L.) Mull. Arg. Leaf Extract With Enhanced Antibacterial, Antioxidant, and Tyrosinase Inhibitory Activities
Source: Int J Microbiol. 2026 Jul 30;2026:3943779. doi: 10.1155/ijm/3943779 (PMC13424607; doi:10.1155/ijm/3943779)
Supplement: Supplementary file 1 — Supporting Information Additional supporting information can be found online in the Supporting Information section. Figure S1: Agar well diffusion assay results showing the antibacterial activity of Macaranga tanarius extract (MTE) against 17 clinical Staphylococcus aureus isolates from atopic dermatitis patients, including mecA‐positive strains. Details are provided in the Supporting Information Data. Figure S2: UV–Vis spectral results showing the catalytic degradation efficiency of Macaranga tanarius–synthesized silver nanoparticles (MT‐AgNPs) against Congo red in the presence of NaBH4. Details are provided in the Supporting Information Data. [file IJM-2026-3943779-s001.docx]

**Supplementary data:**


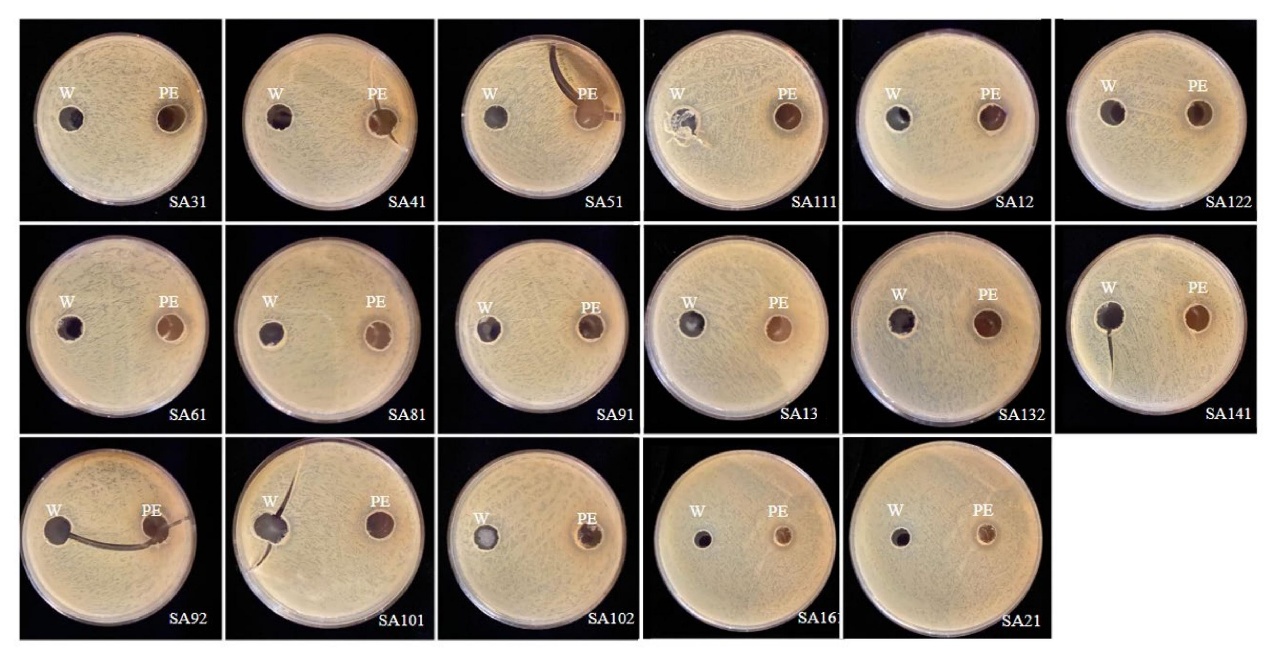


**Fig. S1. Antibacterial activity of Macaranga tanarius extract (MTE) against clinical Staphylococcus aureus isolates from atopic dermatitis (AD) patients.**
Seventeen clinical S. aureus strains were tested using the agar well diffusion assay. Compared to the sterile water control (left well; W), clear inhibition zones were consistently observed around wells containing MTE (PE: plant extract), indicating its antibacterial activity. Notably, this included strains SA12 and SA13, both mecA-positive and thus potentially methicillin-resistant S. aureus (MRSA). The consistent inhibitory effect across all isolates suggests that MTE exhibits broad-spectrum antibacterial potential.

**
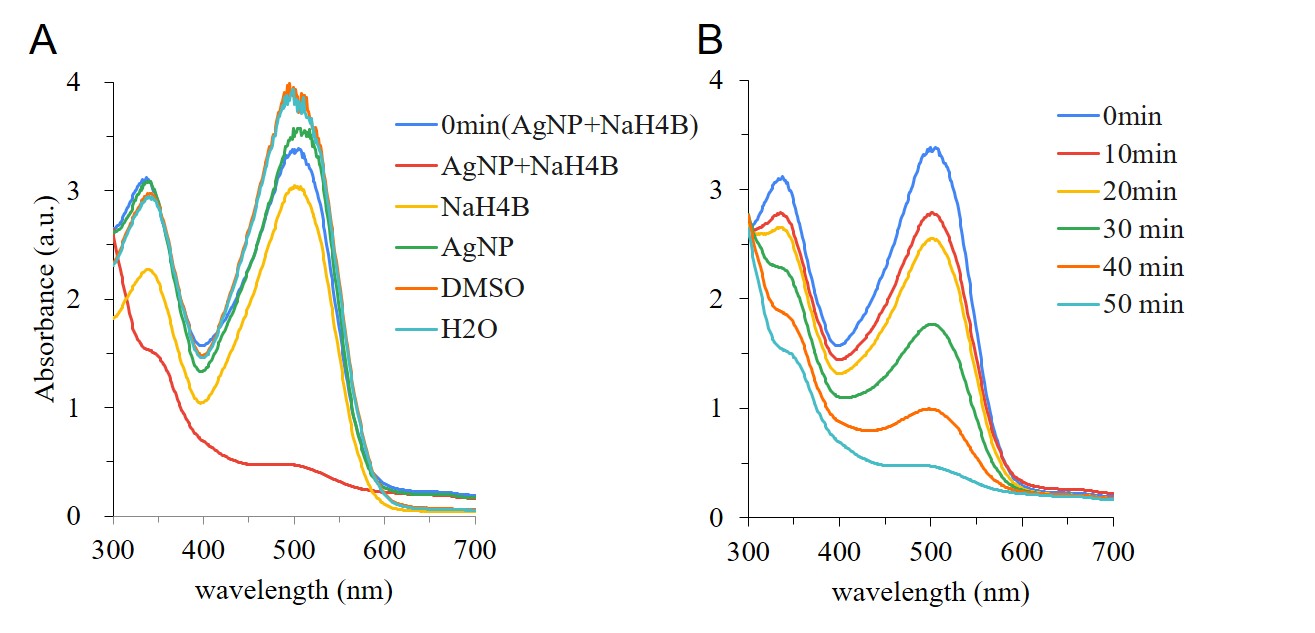
**

**Fig. S2. Catalytic degradation of Congo red by MT-AgNPs.** **(A)** UV-vis absorption spectra of Congo red under various control conditions compared to the experimental group. The spectra show no significant degradation in control groups lacking either the catalyst (NaBH₄ only, DMSO, H₂O) or the reducing agent (AgNP only). Significant decolorization is observed only in the complete reaction system (AgNP + NaBH₄). **(B)** Time-dependent UV-vis spectral changes of Congo red catalyzed by MT-AgNPs in the presence of NaBH₄. The absorption intensity was monitored at 10-minute intervals from 0 to 50 min, indicating progressive degradation of the dye.
